# Predicting Dropout From Organized Football: A Prospective 4-Year Study Among Adolescent and Young Adult Football Players

**DOI:** 10.3389/fspor.2021.752884

**Published:** 2022-01-17

**Authors:** Nico W. Van Yperen, Laura Jonker, Jan Verbeek

**Affiliations:** ^1^Department of Psychology, University of Groningen, Groningen, Netherlands; ^2^Centre for Human Movement Sciences, University Medical Centre, University of Groningen, Groningen, Netherlands; ^3^XOET, Bern, Netherlands; ^4^Royal Netherlands Football Association, Zeist, Netherlands

**Keywords:** dropout, sport attrition, withdrawal, enjoyment, motivation, soccer, turnover

## Abstract

Previous studies have shown that enjoyment is one of the key predictors of dropout from organized sport, including organized football. However, prospective studies, particularly studies focused on long-term dropout, are largely lacking. Drawing on the basic principles of interdependence theory, in the present prospective study among 1,762 adolescent and young adult football players (27.1% women, mean age 17.74 years, SD = 1.35), we tested the predictive value of sport enjoyment, perceived alternatives, and restraining forces on football players' short-term (6 months) and long-term (4 years) dropout from organized football. As anticipated, the results of the logistic regression and follow-up analyses indicate that players' enjoyment was the main predictor of (short-term and long-term) dropout. In addition, relative to remainers, dropouts perceived more alternatives in terms of other sports, had fewer family members involved in their football club, and were older at the time they started playing organized football. We conclude that particularly measures aimed at enhancing sport enjoyment may prevent players from dropping out from organized football in both the short and long term. In addition, dropout rates may be reduced by attracting and engaging youth at a very young age (from 6 years), and their siblings, parents, and other family members as well.

## Introduction

Football (referred to as “soccer” in the USA) is by far the most popular sport in the world. This top-ranked position is based on 15 different criteria, including global fan base and audience, presence in social media, number of countries in which the sport is popular, and the number of amateur players in the world (Sourav, 2021). A count by the Fédération Internationale de Football Association (FIFA, 2006) indicated that approximately 265 million people in more than 200 countries play football regularly. Participation in sports such as football, and physical activity in general, have been associated with a series of physical, psychological, social, and health benefits among youth, particularly adolescents (e.g., Janssen and LeBlanc, 2010; Eime et al., 2013; Gísladóttir et al., 2013; Rodriguez-Ayllon et al., 2019; Weiss, 2019). Youth sport additionally provides valuable lessons and life skills which can be transferred to other life settings and are useful later on in life (e.g., Dohme et al., 2019).

It is therefore unfortunate that across countries and sports, dropout rates from organized sport are quite high (10–40%), particularly among girls and adolescents (e.g., Patriksson, 1988; Balish et al., 2014; Crane and Temple, 2015; Møllerløkken et al., 2015; Wagnsson et al., 2021). Across organized sports, but especially in the world's major sport, a high dropout rate represents many millions of (youth) participants. Furthermore, football (like other team sports) requires a minimum of players in order to compete. Accordingly, the dropout of individuals also puts the existence of the team as a whole at risk, which may lead team members to decide to quit as well (cf. Schlesinger et al., 2018). Dropout from organized football (and organized sports in general) is therefore worth consideration for the well-being of young people. For example, youth who dropped out of organized sports, including football, reported lower physical activity, greater body fat, and higher depressive symptomatology at age 20 than those who continued participation (Howie et al., 2016). Furthermore, research has demonstrated that sports participation in childhood and adolescence is positively related to participation in sports in adulthood (Lee et al., 2018; Biagi Batista et al., 2019).

Remarkably, however, prospective studies, and particularly studies that focus on predicting long-term dropout (>3 years), are largely lacking (Guzmán and Kingston, 2012; Gardner et al., 2017). By gaining empirical insight into factors that can explain actual dropout, and long-term dropout in particular, effective and timely measures can be developed and applied to proactively reduce dropout rates among youth. In the present study, we focused on adolescents and young adults (16–21 years of age). The Royal Netherlands Football Association has observed a relatively high dropout rate from football clubs (> 50% over a period of 4 years) within this age group. Indeed, the junior-to-senior transition has frequently been cited as the most difficult within-career transition in athletes' careers (Drew et al., 2019). Hence, the aim of the current study was to extend prior research by using a prospective design to examine factors associated with young players' actual dropout from organized football, both short-term (6 months) and long-term (4 years).

## Predicting Dropout From Organized Football

In the sport domain, fun or sport enjoyment has been found to be a key factor in the explanation of dropout (e.g., Butcher et al., 2002; Crane and Temple, 2015; Visek et al., 2015; Scanlan et al., 2016; Gardner et al., 2017; Schlesinger et al., 2018; Witt and Dangi, 2018). Enjoyment is defined as the positive affective response to the sport experience that reflects generalized feelings of joy (Scanlan et al., 2016). Obviously, the stronger the positive affective response to a sport experience, the stronger the intention to stay and to persist in the sport activity.

However, sport enjoyment is not the sole basis for dropping out from football. Drawing on the basic principles of interdependence theory (e.g., Kelley and Thibaut, 1978; Schmidt and Stein, 1991), and more specifically, the models developed by Rusbult and Farrell (1983) and Scanlan et al. (2016), we anticipated that the availability of attractive and valued alternatives and costs of staying or quitting, may instigate the decision to leave, even when enjoyment is high (cf., Van Yperen, 1998; Butcher et al., 2002; Steel and Lounsbury, 2009; Schlesinger et al., 2018). As perceived alternatives we specifically focused on doing other sports and playing pickup football that conflict with continued participation in organized club football or staying with the club. In contrast, the decision to stay may be strengthened when psychological and tangible costs of quitting increase. For example, the years of investment in their football club may be perceived as a loss when players consider to cancel their football club membership. Similarly, when family members are also involved in the club, the social costs of leaving the club may be perceived as (too) high. Accordingly, our hypothesis was that dropout would be a function of players' (1) sport enjoyment, (2) alternative options (i.e., involvement in another sport and playing pickup football), and (3) restraining forces (i.e., involvement of family members in the club and years of investment).

The demographic variables age and sex are relevant factors to consider in this context (e.g., Butcher et al., 2002; Guzmán and Kingston, 2012; Balish et al., 2014; Møllerløkken et al., 2015; Gardner et al., 2017). Specifically, as young people progress through adolescence, physical activity levels tend to decline (Dumith et al., 2011) and participation in organized sport drops (Zimmermann-Sloutskis et al., 2010; Eime et al., 2016). Hence, among youngsters, dropout rates would be positively associated with age. Furthermore, because of the different socially constructed meanings often associated with sport participation (McCarthy et al., 2008; Cairney et al., 2015; Crane and Temple, 2015), dropout rates would be higher among girls than among boys.

## Method

### Participants

Participants were active members of a Dutch football club in the season 2016/2017, and accordingly, members of the Royal Netherlands Football Association. A total of 1,762 players had complete datasets. The percentage of women (27.1%) indicates an overrepresentation of women in the current sample, χ^2^(1) = 153.4, *p* <0.001 (the actual percentage of female members was 16.2%). The age range was 16–21, with a mean of 17.74 years (SD = 1.35). A large majority (82.5%) indicated that they also played pickup football, and only a minority (22.2%) also practiced a sport other than football. Some 47.6% indicated that at least one other family member was also a member of their football club, and most players (74.2%) started playing organized football as members of an Under 8 team (45.7%) or Under 6 team (28.5%).

After 4 years, a majority (72.5%) was still a member of their football club (“remainers”); 4.0% had canceled their membership within 6 months (short term), and 23.5% within 4 years (long term). After 4 years, the actual dropout from organized football was 55.9%, indicating that the number of dropouts (27.3%) was underrepresented in our sample, χ^2^(1) = 575.5, *p* < 0.001.

### Procedure

The study was initiated by the Royal Netherlands Football Association (1,231,561 members in 2016), who were particularly interested in the age group 16–21; that is, players who had to deal with the junior-to-senior transition, which has frequently been cited as the most difficult within-career transition in athletes' careers (Drew et al., 2019). Ethical approval was obtained from the university's ethical committee. On behalf of the Royal Netherlands Football Association and the University of Groningen, a research assistant sent an email to all players in the age range between 16 and 21 who were registered as players (*n* = 41,870): “*To make football as much fun as possible, we would like to know what your reasons are for playing football. We kindly ask you to follow the link and fill out the survey.”* We indicated that participation in the study was voluntary and confidential, that they could stop their participation any time, and that all data would be anonymized. An email address of the football association was provided for questions or comments.

In April 2017 (T_0_), about a month before the end of the competition season, a total of 2,395 (5.7%) had clicked on the link to the digital survey. The participants who were included in the final sample (*n* = 1,762) had a complete dataset and had provided explicit consent prior to the processing of their personal data for scientific research purposes. The participants who were excluded had indicated at the end of the survey that they (1) had not answered all the questions honestly, (2) should have been more accurate when answering all the questions, or (3) had not read all the questions carefully (e.g., Meade and Craig, 2012; Cheung et al., 2017).

By checking the membership status from the participants that completed the survey at T_0_, short-term dropout (i.e., cancellation of their membership) was assessed 6 months later (T_1_, November 2017). Long-term dropout was assessed by checking membership status of the participants 4 years later (T_2_, February 2021).

### Measures

Dropout from organized football was defined as the cancellation of one's membership of the Royal Netherlands Football Association. This was assessed through membership status after 6 months (short-term) and after 4 years (long-term).

Enjoyment was defined as the positive affective response to football that reflects generalized feelings of joy and was measured using three items (Van Yperen, 1998; Scanlan et al., 2016): (1) I enjoy playing football; (2) I have fun playing football; (3) I like playing football. Each item was followed by a Likert scale ranging from *never* (1) to *always* (7). Scale scores were obtained by averaging the scores on the individual items. Cronbach's alpha was 0.89.

Perceived alternatives were represented by two dichotomous variables that indicated whether the respondent (Alt1) only played football (*yes* = 0, *no, other sports as well* = 1), and (Alt2) only played organized club football (*yes* = 0, *no* = 1).

Restraining forces were represented by two variables: (RF1) the involvement of family members in the club (*no* = 0, *yes* = 1), and the following question (RF2): When you started playing organized club football, which team did you join? Response categories ranged from 1 = *Under 6*, to 7 = *Under 18*, and 8 = *Senior (*> *18 years of age)*.

## Results

### Descriptive Data

Table 1 presents the means, standard deviations, and correlations between all variables. In general, the correlations between the variables were not very high. Sport enjoyment was moderately correlated with playing pickup football as well (Alt2). The highest correlation was between sex and starting playing organized club football at a younger age (RF2). That is, relative to girls (*M* = 3.23, SD = 1.64), boys (*M* = 1.95, SD = 1.17) were younger at the time they started playing organized football, *t*_(1, 760)_ = 18.09, *p* < 0.001.

**Table 1:** Means, standard deviations, and correlations (*n* = 1,762).

	**M**	**SD**	**2**	**3**	**4**	**5**	**6**	**7**
Enjoyment	6.34	0.82	−0.05	0.25	0.03	−0.09	−0.12	0.12
Alt1: Only football	0.22	0.42	-	−0.01	−0.02	0.03	0.04	0.03
Alt2: Org. football	0.82	0.38		-	0.02	−0.15	−0.19	0.23
RF1: Family	0.48	0.50			-	−0.01	0.01	−0.05
RF2: First team	2.30	1.43				-	0.09	−0.40
Age	17.74	1.35					-	−0.00
Sex	0.73	0.44						-

*Correlations >0.09 are significant at p < 0.001*.

*Alt1, Only played football (no other sport); Alt2, Only played organized football; RF1, Involvement of family members in the club; RF2, Age group when started playing football for an official club; Sex, female = 0, male = 1*.

### Testing the Hypotheses

Our hypothesis was that dropout would be a function of players' (1) sport enjoyment, (2) alternative options (i.e., involvement in another sport and playing pickup football), and (3) restraining forces (i.e., involvement of family members in the club and years of investment). To test this hypothesis, a logistic regression analysis was conducted with dropout (short-term vs. long-term vs. remainer) as the dependent variable. The numeric predictor variables (assessed at T_0_) were enjoyment, starting team (restraining force), and age, and the nominal predictor variables were perceived alternatives (only played football and only played organized football), the involvement of family members in the club (restraining force), and sex. The analysis focused on comparing the short-term and the long-term dropout rates, with the remainers as the reference category.

As can be seen in Table 2, the logistic model provides a good fit to data. The statistical significance of individual regression coefficients (β) was tested using the Wald chi-square statistic. Also in the present study, sport enjoyment appears to be a key factor in explaining dropout, and actually the only predictor of short-term dropout. An additional analysis of variance revealed that relative to the remainers (*M* = 6.43, SD = 0.75), sport enjoyment was much lower among the short-term dropouts (*M* = 5.63, SD = 1.15) and lower among long-term dropouts (*M* = 6.18, SD = 0.89), *F*_(2, 1759)_ = 43.52, *p* < 0.001. Bonferroni *post-hoc* tests indicated that these differences between the three groups were significant (*p*s < 0.001).

**Table 2:** Logistic regression results with dropout from organized football (remainers vs. short-term dropouts vs. long-term dropouts) as the dependent variable.

**Predictor**	**ß**	**SE ß**	**χ^2^**	**Exp(B) (odds ratio)**	**95% CI Exp(B)**	
					**Lower bound**	**Upper bound**
**Remainers vs. short-term**						
Intercept	0.71	1.85	0.15			
Enjoyment	−0.79**	0.12	42.38**	0.46	0.36	0.58
Alt1: Only football	0.49	0.28	3.19	1.63	0.95	2.80
Alt2: Org. football	−0.55	0.30	3.27	0.58	0.32	1.05
RF1: Family	−0.38	0.26	2.15	0.69	0.42	1.14
RF2: First team	0.19	0.08	5.29	1.21	1.03	1.42
Age	0.05	0.09	0.30	1.05	0.88	1.25
Sex	0.43	0.32	1.76	1.53	0.82	2.87
**Remainers vs. long-term**						
Intercept	3.46	0.96	13.07			
Enjoyment	−0.31**	0.07	18.93**	0.73	0.64	0.84
Alt1: Only football	0.41*	0.13	9.37*	1.51	1.16	1.96
Alt2: Org. football	−0.39*	0.16	6.21*	0.68	0.50	0.92
RF1: Family	−0.31*	0.12	7.01*	0.73	0.58	0.92
RF2: First team	0.17**	0.04	16.17**	1.18	1.09	1.28
Age	−0.14*	0.05	9.98*	0.87	0.79	0.95
Sex	−0.19	0.14	1.85	0.83	0.63	1.09
-2 Log likelihood	1637.41
Model Chi-Square	139.05** (*df* = 14)
Classification accuracy	72.9%

*p < 0.01, **p < 0.001.

*Alt1, Only played football (no other sport); Alt2, Only played organized football; RF1, Involvement of family members in the club; RF2, Age group when started playing football for an official club; Sex, female = 0, male = 1*.

Table 2 also shows that perceived alternatives and restraining forces predicted additional variance of long-term dropout. Specifically, relative to the long-term dropouts (27.5%), only 20% of the remainers indicated that they did another sport as well (Alt1), χ^2^(1, *N* = 1,691) = 10.26, *p* = 0.001. Unexpectedly, however, more than the long-term dropouts (76.3%), the remainers (85.3%) perceived more alternatives *within* the football context (Alt2), χ^2^(1, *N* = 1,691) = 17.82, *p* < 0.001. That is, the remainers played more pickup football, which likely reflects their higher levels of enjoyment (*r* = 0.25, see Table 1). The differences between the remainers and the short-term dropouts were in the same direction, but not significant.

As anticipated, also the restraining forces explained additional variance of long-term dropout (see Table 2). Specifically, our findings suggest that the involvement of family members in their club (RF1) may have prevented the remainers from quitting. An additional chi-square test, χ^2^(1, *N* = 1,691) = 7.73, *p* = 0.005, revealed that the percentage of family members involved in the club was higher among the remainers (49.9%) than among the long-term dropouts (42.0%). Furthermore, an additional analysis of variance revealed a link between dropout and years of investment, *F*_(2, 1759)_ = 17.60, *p* < 0.001. Specifically, Bonferroni *post-hoc* tests indicated that relative to the long term-dropouts (*M* = 2.62, SD = 1.58, *p* < 0.001), the remainers (*M* = 2.17, SD = 1.35) tended to have been in a younger age group when they joined a football club (*p* < 0.001). Also with regards to the restraining forces, the differences between the remainers and the short-term dropouts were in the same direction, but not significant.

Finally, follow-up analyses of variance indicated that the effect of age [*F*_(2, 1759)_ = 4.42, *p* = 0.01] could be ascribed to the short-term dropouts (*M* = 18.11, SD = 1.15) who were (marginally) older than the long-term dropouts (*M* = 17.62, SD = 1.32, *p* = 0.01) and the remainers (*M* = 17.76, SD = 1.36, *p* = 0.09).

In sum, the results of the logistic regression and follow-up analyses indicate that the dropouts enjoyed football less, perceived more alternatives in terms of other sports, and had fewer family members involved in their football club. Dropouts' lower levels of sport enjoyment may also be reflected in their lower rates of involvement in pickup football. In addition, dropouts were somewhat older at the time they started playing organized football (i.e., dropouts had less invested in football).

## Discussion

The findings of this first prospective study on long-term dropout from organized football evidenced the key role of enjoyment in the sport experiences of youth (e.g., Butcher et al., 2002; Crane and Temple, 2015; Visek et al., 2015; Gardner et al., 2017; Schlesinger et al., 2018; Witt and Dangi, 2018). Specifically, the results indicate that lack of enjoyment is the main predictor of both short-term (6 months) and long-term (4 years) dropout. These findings suggest that interventions that focus on educating coaches in how to enhance athletes' sport enjoyment have the potential to prevent youngsters to cancel their club membership, and accordingly, reduce dropout rates in organized football (cf. Barnett et al., 1992; García Bengoechea et al., 2004). For example, coaches may learn how to communicate with their athletes in a more need-supportive manner, which is likely to enhance athletes' feelings of enjoyment (e.g., Domville et al., 2019). Cox et al. (2009) demonstrated that athletes' perceptions of how their physical educator cared about and understood them as individuals were important for their enjoyment. Furthermore, a review of 51 autonomy-supportive interventions (including 38 randomized control trials) demonstrated that an autonomy-supportive instruction style had positive effects on desirable outcomes such as enjoyment, intrinsic motivation, and performance (Reeve and Cheon, 2021). Coaches support their athletes' need for autonomy when they—among other things—consider the athletes' perspective, nurture their intrinsic motivational resources, and rely on informational rather than controlling language.

Sport enjoyment is apparently important, but it is not the sole basis for dropout. As expected, the chances of dropout from organized football (in the long term) were lower when football players had more family members involved in their football club and when they were somewhat younger at the time they started playing organized club football. Because boys were clearly younger than girls at the time they started playing organized football (see Table 1), girls and their parents may need some time to overcome social and cultural beliefs that associate sport primarily with masculinity and the male identity (McCarthy et al., 2008; Cairney et al., 2015; Crane and Temple, 2015).

These findings suggest that the decision to continue one's membership of the football club was strengthened by restraining forces, or the psychological and tangible costs of quitting (Rusbult and Farrell, 1983; Schmidt and Stein, 1991; Van Yperen, 1998; Steel and Lounsbury, 2009; Scanlan et al., 2016). We also showed that doing another sport (i.e., an attractive and valued alternative) explained additional variance of dropout from organized football, and long-term dropout in particular. In contrast, playing pickup football was associated with stay behavior rather than leave behavior. An explanation may be that involvement in pickup football reflects higher levels of enjoyment rather than an alternative for organized club football (cf. Côté et al., 2009). Moreover, playing pickup sports may satisfy youth players' need for autonomy, and accordingly, their sport enjoyment (e.g., Ryan and Deci, 2002). When playing pickup football, players (rather than their parents or coaches) pick the teams, make the rules, and call their own fouls. Accordingly, they learn how to collaborate with others, how to work out problems with their teammates and their opponents, and how to make the rules of the game fair for everyone. Altogether, our findings suggest that football clubs may reduce dropout rates not only by enhancing players' sport enjoyment, but also by attracting and engaging youth at a very young age (from 6 years), and their siblings, parents, and other family members as well. That is, organized club football may be considered a family affair, an extension and inclusion of family in which sport and family are intrinsically linked.

### Strengths and Limitations

A main strength of the present research is that we add to the extent literature on dropout by focusing on predicting long-term dropout. Also, our findings suggest that effective interventions should primarily focus on enhancing football players' enjoyment, for example, by supporting their basic psychological needs (e.g., Ryan and Deci, 2002) and approach motivation (Van Yperen, 2021).

Balanced against these strengths, limitations need to be acknowledged. First, our final sample comprised a total of 1,762 football players. Although this is quite an impressive sample size, it is only 4% of the 41,870 football players aged between 16 and 21 who were registered as members of the Royal Netherlands Football Association. In this final sample, the number of females was somewhat overrepresented, and the number of dropouts in our sample was lower than the actual number registered by the Royal Netherlands Football Association.

Second, based on theory and previous findings (e.g., Rusbult and Farrell, 1983; Schmidt and Stein, 1991; Van Yperen, 1998; Scanlan et al., 2016), we included sport enjoyment and specific perceived alternatives and restraining forces as predictor variables. For practical reasons, however, many other variables that may be relevant as well, were not included (e.g., Fraser-Thomas et al., 2008; Balish et al., 2014; Visek et al., 2015). Examples are physical factors such as injuries (Brenner, 2007; Crane and Temple, 2015) and psycho-social factors such as the relationship with parents, peers, trainers, and teammates (Fraser-Thomas et al., 2008; Schlesinger et al., 2018), relative age (Delorme et al., 2010), success and goal achievement (Schlesinger et al., 2018), perceived disadvantage (Van Yperen, 1997), and burnout (Schmidt and Stein, 1991; Gustafsson et al., 2011). Furthermore, particularly during adolescence, athletes face life situations related to work, education, and interpersonal relationships which require their attention (Rottensteiner et al., 2013). In the present study, we did not assess the perceived availability of such attractive and valued alternatives outside sports that may instigate the decision to cancel one's membership of the club. Relatedly, we do not know whether the participants who dropped out of organized football, dropped out of sport completely (and permanently). Actually, for our dropout data, we relied on records from the Royal Netherlands Football Association that only indicated whether or not the respondents canceled their membership of their football club. The chances are good that (at a later point in time) they decided to take up a new sport, and accordingly, gained their physical, psychological, social, and health benefits through another sport (e.g., Patriksson, 1988; Butcher et al., 2002).

Finally, in the present study, we focused on identifying and assessing correlates of youth sport attrition (cf. Balish et al., 2014). Important to note, however, is that predictors of dropout behavior do not act independently of each other (e.g., Schlesinger et al., 2018). Most likely, dropping out from football is a product of dynamic interactions between multiple components (Den Hartigh et al., 2018; Van Yperen, 2021), including the factors identified in the present research. Furthermore, such processes and pathways are typically idiosyncratic and non-linear (e.g., Bjørndal et al., 2016). Accordingly, in future research, dropout may be conceptualized from a dynamical systems perspective where dropout is a function of dynamic person-environment interactions (cf. Den Hartigh et al., 2018).

## Conclusion

The main finding of our prospective study is that enjoyment is the main predictor of (short-term and long-term) dropout. This finding suggests that particularly measures aimed at enhancing sport enjoyment may prevent players from dropping out from organized football in both the short and long term. In addition, dropout rates may be reduced by attracting and engaging youth at a very young age (from 6 years), and their siblings, parents, and other family members as well.

## Data Availability Statement

The raw data supporting the conclusions of this article will be made available by the authors on request.

## Ethics Statement

The studies involving human participants were reviewed and approved by Faculty of Behavioral and Social Sciences, University of Groningen, the Netherlands. The participants had provided explicit consent prior to the processing of their personal data for scientific research purposes.

## Author Contributions

NWVY conceived the study, developed the theoretical framework, supervised the data collection, analyzed the data, and wrote the manuscript. LJ conceived the study, supervised the data collection, and provided feedback on the manuscript. JV supervised the data collection and provided feedback on the manuscript. All authors contributed to the article and approved the submitted version.

## Conflict of Interest

The authors declare that the research was conducted in the absence of any commercial or financial relationships that could be construed as a potential conflict of interest.

## Publisher's Note

All claims expressed in this article are solely those of the authors and do not necessarily represent those of their affiliated organizations, or those of the publisher, the editors and the reviewers. Any product that may be evaluated in this article, or claim that may be made by its manufacturer, is not guaranteed or endorsed by the publisher.
